# Salivary microbiome in patients undergoing hemodialysis and its associations with the duration of the dialysis

**DOI:** 10.1186/s12882-020-02009-y

**Published:** 2020-09-29

**Authors:** Xiaobo Duan, Xiaolei Chen, Megha Gupta, Dutmanee Seriwatanachai, Hanxiao Xue, Qiuchan Xiong, Tong Xu, Dan Li, Anchun Mo, Xi Tang, Xuedong Zhou, Yuqing Li, Quan Yuan

**Affiliations:** 1 State Key Laboratory of Oral Diseases & National Clinical Research Centre for Oral Diseases, West China Hospital of Stomatology, Sichuan University, No. 14, Section 3, Renmin South Road, Chengdu, 610041 Sichuan China; 2 Department of Nephrology, West China Hospital, Sichuan University, Chengdu, China; 3 Department of Preventive Dental Sciences, Division of Pedodontics, College of Dentistry, Al-Showajra Academic Campus, Jazan University, Jazan, Kingdom of Saudi Arabia; 4 Department of Oral Biology, Faculty of Dentistry, Mahidol University, Bangkok, Thailand; 5 Department of Oral Implantology, West China Hospital of Stomatology, Sichuan University, Chengdu, China

**Keywords:** Saliva, Oral microbiome, Hemodialysis, Periodontal disease

## Abstract

**Background:**

Chronic kidney disease (CKD) patients, especially those with end stage renal disease (ESRD) undergoing hemodialysis (HD), exhibit high prevalence of periodontitis. This cross-sectional study aimed to investigate the periodontal status of HD patients and its relationship with salivary microbiome.

**Methods:**

One hundred eight HD patients and one hundred healthy control individuals were recruited. They were subjected to periodontal examination followed by saliva samples collection for 16S rRNA gene sequencing.

**Results:**

The HD patients were with worse periodontal health status, and exhibited higher salivary microbial diversity and lower richness. The periodontal pathogens were significantly enriched in the HD patients. The inferred functional analyze showed microbes enriched in the HD patients were mainly related to metabolism. Despite the periodontal status and overall structure of the microbiome were not significantly altered as the HD duration prolonged, the abundance of *Lachnospiraceae [G-2] sp.* |*HMT_096*| is positively correlated with the duration of HD and the community periodontal index (CPI). Five OTUs (operational taxonomic units) belonging to the phyla *Firmicutes* were enriched as the duration prolonged, and four OTUs originated from the phyla *Proteobacteria* were negatively related with the CPI index. ESRD patients undergoing HD exhibited microbiota structural, compositional and functional differences compared with the healthy controls. And the species changed as the duration of hemodialysis prolonged.

**Conclusions:**

End stage renal disease changes salivary microbiome and is a risk factor for oral dysbiosis.

## Background

Chronic kidney disease (CKD) is characterized by a presence of injury and/or a progressive loss of renal function over a period of months or years culminating with end stage kidney disease (ESKD) [1]. Due to serious systemic sequelae, CKD has emerged as a major public health, the global burden of which has increased substantially in recent years [2]. When the functional capacity of the kidney decreases below 5–10% of the normal efficiency, hemodialysis (HD), the most common form of the renal replacement therapy, can improve the long-term survival of patients with ESKD [3].

CKD is a risk factor for chronic periodontitis [4–6]. For patients with ESRD, especially, worse oral hygiene and higher prevalence of periodontal diseases were reported [7–9]. Moreover, the risks of oral problem increase as the renal failure progresses [10].

A complex community of bacteria, archaea, protozoa, fungi and viruses colonize our human body, which encompasses 10 times more cells and 100 times more genes than the host [11]. The oral microbiome can damage the dynamic balance between health and disease, locally and systemically [12]. The microbiome in saliva sheds from adhering microbial niches on various intraoral surfaces and appears to be the representative of the oral microbiome integrity. Salivary microbiome has a critical role on the genesis of oral diseases, mainly including dental caries, periodontitis and peri-implantitis [13–15]; non-oral diseases such as cardiovascular disease [16], cancer [17, 18] and low birth weight neonate [19] are also somewhat related to the dysbiosis of salivary microbiome.

Gut dysbiosis has already been well described in CKD patients [20, 21] and ESRD patients undergoing HD [22]. The oral cavity is the primary entry site of the gastrointestinal, additionally, and the oral pathogenic microbes has been described to induce the dysbiosis of gut microbiota in an animal model [23]. Previous studies, using microbiological methods with limited scope, have found that the contents of periodontal pathogens increased in ESRD patients as compared to non-CKD controls [24, 25]. However, until now, very few studies analyzed the salivary microbiota compositions in HD patients.

In this study, we aimed to compare the periodontal status and saliva microbiota between HD patients and healthy persons, and further investigated the association between the salivary microbiome and the duration of HD treatment. These results enrich our understanding of the relationship between general disease state, oral microbiome changes and oral health status.

## Methods

### Study population

This study was approved by the Institutional Review Board of the Sichuan University Hospital. The case group comprised 108 patients undergoing HD three times per week at the West China Hospital, Sichuan University, Chengdu, China. They were in stable clinical conditions for at least 3 months. Patients with diabetic nephropathy or kidney transplantation, or patients who had been on absorbable antibiotics or undergoing periodontal disease treatment within the preceding 3 months or have smoking habits were excluded. 100 healthy matched subjects were recruited as controls. All participants gave a written informed consent before participation, and the study was carried out in accordance with those ethical standards in the Declaration of Helsinki.

### Oral examination

One trained dentist performed all the clinical measurements. The Community Periodontal Index (CPI) was used to determine the oral periodontal health status, with the score of 6 sites for 10 World Health Organization index teeth [26]. The number of decayed, missing, and filled teeth (DMFT) was assessed according WHO guidelines [27]. To achieve a high quality of the data, an examiner repeatability exercise was undertaken prior to the initiation of the study. The kappa score for intra-examiner reliability was 0.94.

### Saliva collection and DNA isolation

After the oral examination, saliva samples were collected by the same dentist. All participates were instructed not to eat or brush their teeth at least 2 h before sampling. A Salivette (Sarstedt, GER) collection swab with hard pack was used for the saliva collection and storage. Whole unstimulated saliva was collected using the swab in floor of the mouth. The swab was placed back into the collection tube, and stored at − 80 °C before subsequent analysis. Total DNA was isolated from saliva using the QIAamp DNA micro Kit (QIAGEN Sciences, MD, USA) per the manufacturer’s recommendations, with minor modification by adding an extra lysozyme (3 mg ml^− 1^, 1.5 h) treatment step to lyse the bacterial cell.

### 16S rRNA gene amplification and sequencing

Total DNA was extracted from saliva using the QIAamp DNA micro Kit (QIAGEN Sciences, MD, USA) per the manufacturer’s recommendations, with minor modification by adding an extra lysozyme (3 mg ml^− 1^, 1.5 h) treatment step for lysing the bacterial cell. The V3-V4 regions of the bacterial 16S rRNA gene were PCR amplified using barcoded 338F and 806R. Amplicons were extracted from 2% agarose gels and purified using the AxyPrep DNA Gel Extraction Kit (Axygen Biosciences, Union City, CA, USA) and quantified using QuantiFluor™ -ST (Promega, USA). Purified amplicons were pooled in equimolar and paired-end sequenced (2 × 250) on an Illumina MiSeq platform (Illumina, San Diego, CA) according to the standard protocols.

### Sequence information and data analysis

The raw fastq files were first quality-filtered by Trimmomatic and then merged by FLASH. OTUs were clustered by 97% cutoff using UPARSE (version 7.1). To determine the species name and HOT (Oral Taxon ID), sequences were characterized based on the HOMD database. The relative abundance of each bacterial taxon was calculated and typically presented as “Mean ± SD”. The core microbiome of salivary community was analyzed based on previous study [28]. Mann-Whitney U-test was used to compare the differences between the two groups (*P* < 0.05), and Kruskal-Wallis test was used for comparation among three groups (*P* < 0.05). X^2^ test or Fisher’s exact test was used to compare the categorical variables in the contingency tables (*P* < 0.05). Principal co-ordinates analysis (PCoA) and supervised Partial least squares-discriminant analysis (PLS­DA) analysis were assessed using the Simca­P software (version 13.0, Umetrics, Umea, Sweden). Linear discriminant analysis (LDA) effect size (LEfSe) method was implemented with LEfSe (version 1.0). The functionality of the microbiota was performed using PiCRUST and compared between the two groups. Cytoscape (version 3.6.0) was used to visualize the correlations between the microbiota and duration of HD and other clinical index, with a Spearman’s correlation coefficient (|correlation ρ| > 0.3 or 0.4, and *P* < 0.05).

## Results

### Sociodemographic and Oral health characteristics of the study population

One hundred eight HD patients and one hundred healthy subjects were recruited. The two groups matched in age, gender and body mass indices (BMIs) (Table 1). For the HD group, individuals with CPI 1 (23.0% v. 0.9%) and CPI 2 (39.0% v. 10.2%) were less common, while the percentages of CPI 3 (27.0% v. 41.7%) and CPI 4 (3.0% v. 47.2%) individuals were all significantly higher than those in the healthy controls. Both groups have no individuals with CPI X. There were 45 (41.7%) individuals with moderate periodontitis and 51 (47.2%) individuals with severe periodontitis in the HD group, compared with 27 (27.0%) and 3 (3.0%) in the control group. Namely, the percentage of individuals with CPI 3 and CPI 4 in the HD group was nearly 3 times that in the control group. Meanwhile, the HD group demonstrated greater number of missing teeth compared with healthy group (*P* = 0.013).

**Table 1:** Demographics and Clinical Parameters of all Patients

Characteristics	Groups		*P* value
	Healthy Controls (*N* = 100)	HD patients (*N* = 108)	
**Age [years] (Mean ± SD)**	46.78 ± 14.88	46.19 ± 13.33	0.852
**Male/Female**	62/46	50/50	0.284
**BMI [kg/m**^**2**^**] (Mean ± SD)**	21.24 ± 3.23 (17.21–31.45)	22.44 ± 3.65 (16.22–36.33)	0.074
**Length of HD therapy (Mean ± SD)**	–	3.81 ± 2.87	–
**CPI**			0.000
**0**	8 (8.0%)	0 (0.0%)	0.015
**1**	23 (23.0%)	1 (0.9%)	0.000
**2**	39 (39.0%)	11 (10.2%)	0.000
**3**	27 (27.0%)	45 (41.7%)	0.019
**4**	3 (3.0%)	51 (47.2%)	0.000
**X**	0 (0.0%)	0 (0.0%)	–
**Number of teeth (Mean ± SD)**	27.50 ± 2.1	26.90 ± 2.40	0.025
**Number of Decayed teeth (Mean, range)**	0.62, 0–3	0.85, 0–5	0.579
**Number of Missing teeth (Mean, range)**	0.49, 0–5	1.08, 0–15	0.013
**Number of Filled teeth (Mean, range)**	0.51, 0–5	0.41, 0–4	0.684

“-” = vacancy

CPI: The data are presented as n (%).The categories of CPI were defined as: 0 = healthy gingiva; 1 = bleeding observed directly or by using a mouth mirror after probing; 2 = calculus detected during probing but with all of the black bands on the probe visible; 3 = pocket of 4 to 5 mm (gingival margin within the black band on the probe); 4 = pocket of 6 mm (black band on the probe was not visible); X = excluded sextant (< 2 teeth present)

### Overview of salivary microbial differences among the patients

**Fig. 1:**
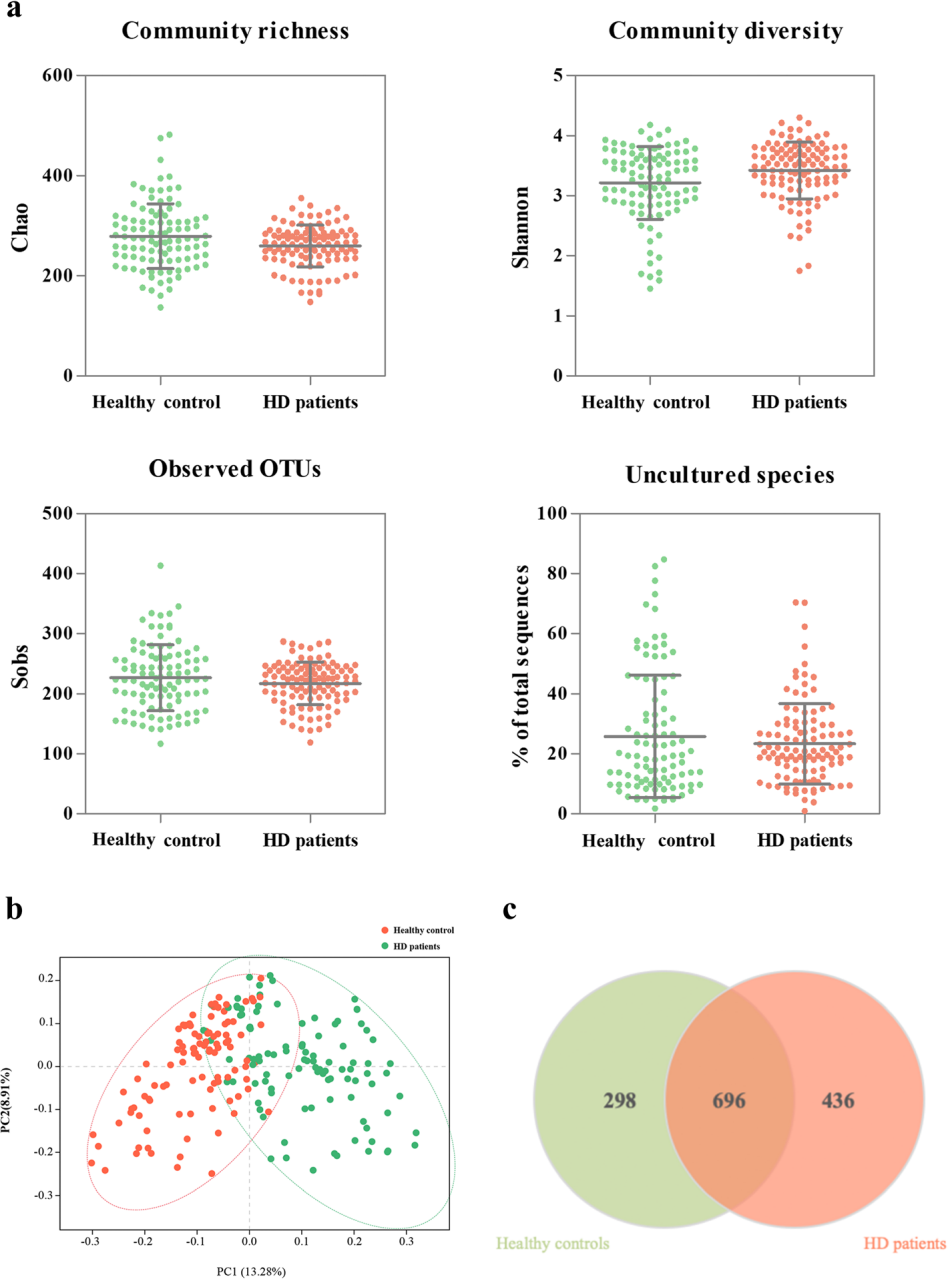
Overview of the salivary microbiota. **a**: Detailed characteristics of alpha diversity (Mean ± SD); **b**: PCoA discriminant analysis plot; **c**: Venn diagram based on OTUs. PCoA: Principal co-ordinates analysis; OTUs: Operational taxonomic units

In total, 29 known phyla and 631 genera were identified, and 1430 OTUs were detected at 3% dissimilarity. To study the richness and diversity of the microbiome from the samples, we compared a series of alpha diversity metrics between the HD patients and the healthy controls, including the Chao index, Shannon index and observed OTUs (Fig. 1a). The HD patients had a lower richness estimator (Chao, *P* = 0.012), and a higher diversity index (Shannon, *P* = 0.016). Uncultivated phylotypes did not significantly differ between the two groups (*P* = 0.610). We generated PCoA plots to investigate the relationship of the composition. The unweighted UniFrac plot showed a distinct separation of the HD samples and the healthy controls based on PC1 (13.28%) and PC2 (8.91%) (Fig. 1b). Consistently, the dissimilarity tests, Adonis and ANOSIM, also revealed significant difference between the two microbial communities (supplementary Table S1). We constructed a Venn diagram and found that 696 OTUs were common in the two communities, while 298 and 436 OTUs were unique to the salivary microbiome of healthy controls and that of HD patients, respectively (Fig. 1c).

**Fig. 2:**
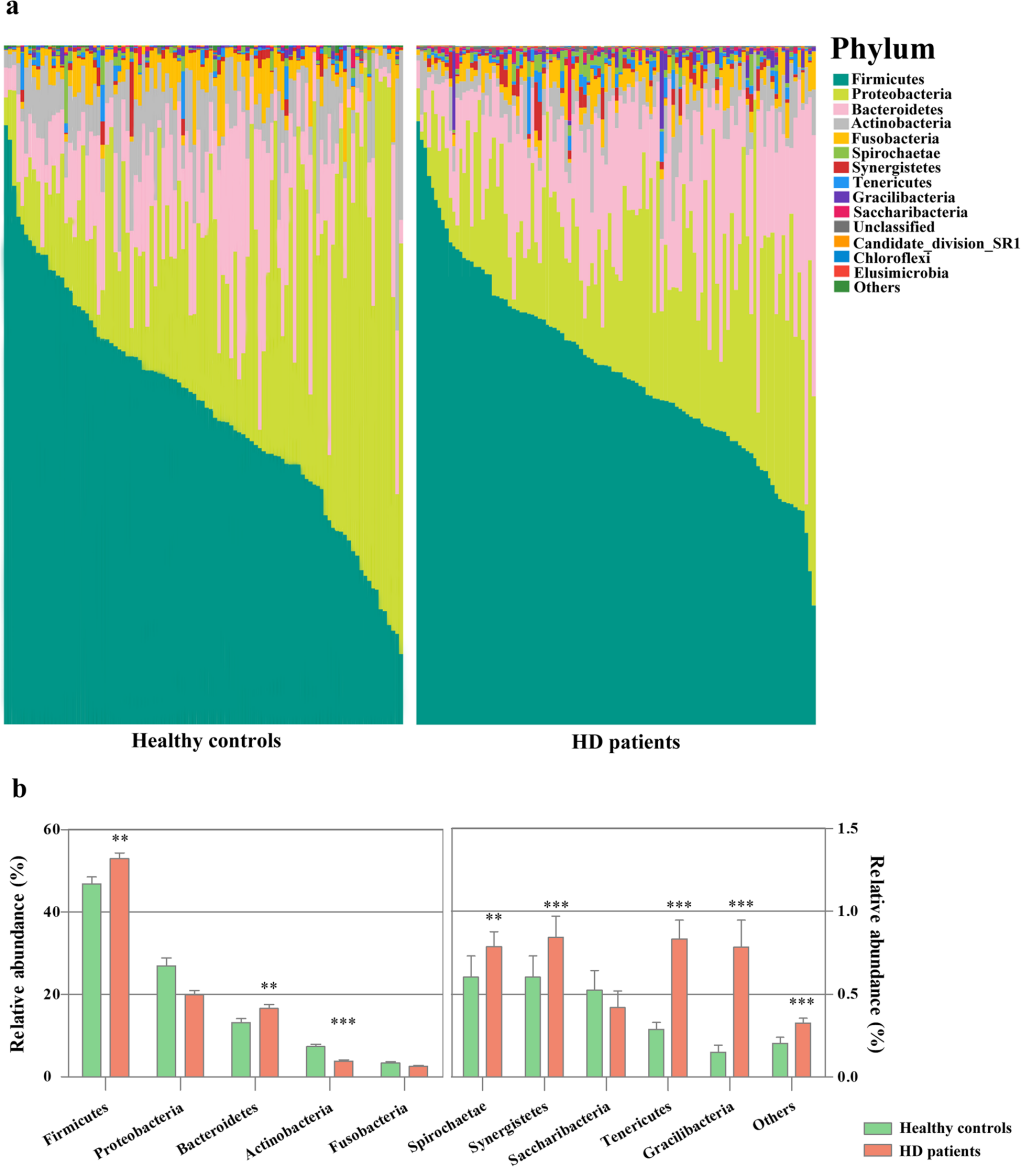
Taxonomic classification of the salivary microbiota at the phylum level. **a**: Relative taxa abundance comparison of the salivary microbiota at the phylum level; **b**: Relative abundance of the top 10 bacterial phyla. The significance of differences between groups was assessed using Mann–Whitney U-test (*P < 0.05, **P < 0.01, ***P < 0.001)

Next, we evaluated the oral microbial community characteristics of the two niches. The taxonomic compositions at the phylum level varied widely between groups (Fig. 2a). *Firmicutes*, *Proteobacteria*, *Bacteroidetes*, *Actinobacteria* and *Fusobacteria* phyla were the dominant phylum (97.66% v. 95.91%) in both groups. The relative abundances of *Firmicutes* (*P* = 0.008), *Bacteroidetes* (*P* = 0.002), *Spirochaetae* (*P* = 0.001), *Synergistetes* (*P* < 0.001), *Tenericutes* (*P* < 0.001), Gracilibacteria (*P* < 0.001), and the total relative abundance of the other 21 phyla (*P* < 0.001) were significantly higher in the HD patients, whereas the abundances of *Proteobacteria* (*P* = 0.074) and *Actinobacteria* (*P* < 0.001) decreased (Fig. 2b). The microbial shift was illustrated in more detail at the genus level. We analyzed the top 30 genera (supplementary Table S2), and the taxa with significant difference were shown in supplementary Table S3, where more than half of these 30 genera were listed. Compared with the healthy cohort, the relative abundances of *Ruminococcaceae* (*P* < 0.001), *Capnocytophaga* (*P* < 0.001), *Porphyromonas* (*P* = 0.001), *Veillonellaceae* (*P* < 0.001), *Granulicatella* (*P* < 0.001) and other 9 genera were significantly enriched in the HD samples. In contrast, *Lautropia* (*P* = 0.031), *Prevotella [G-7]* (*P* < 0.001), *Actinomyces* (*P* < 0.001), *Veillonella* (*P* < 0.001), *Rothia* (*P* = 0.031) and *Leptotrichia* (*P* = 0.027) showed significant overabundance in the healthy controls.

### Changes of the Core microbiota

**Fig. 3:**
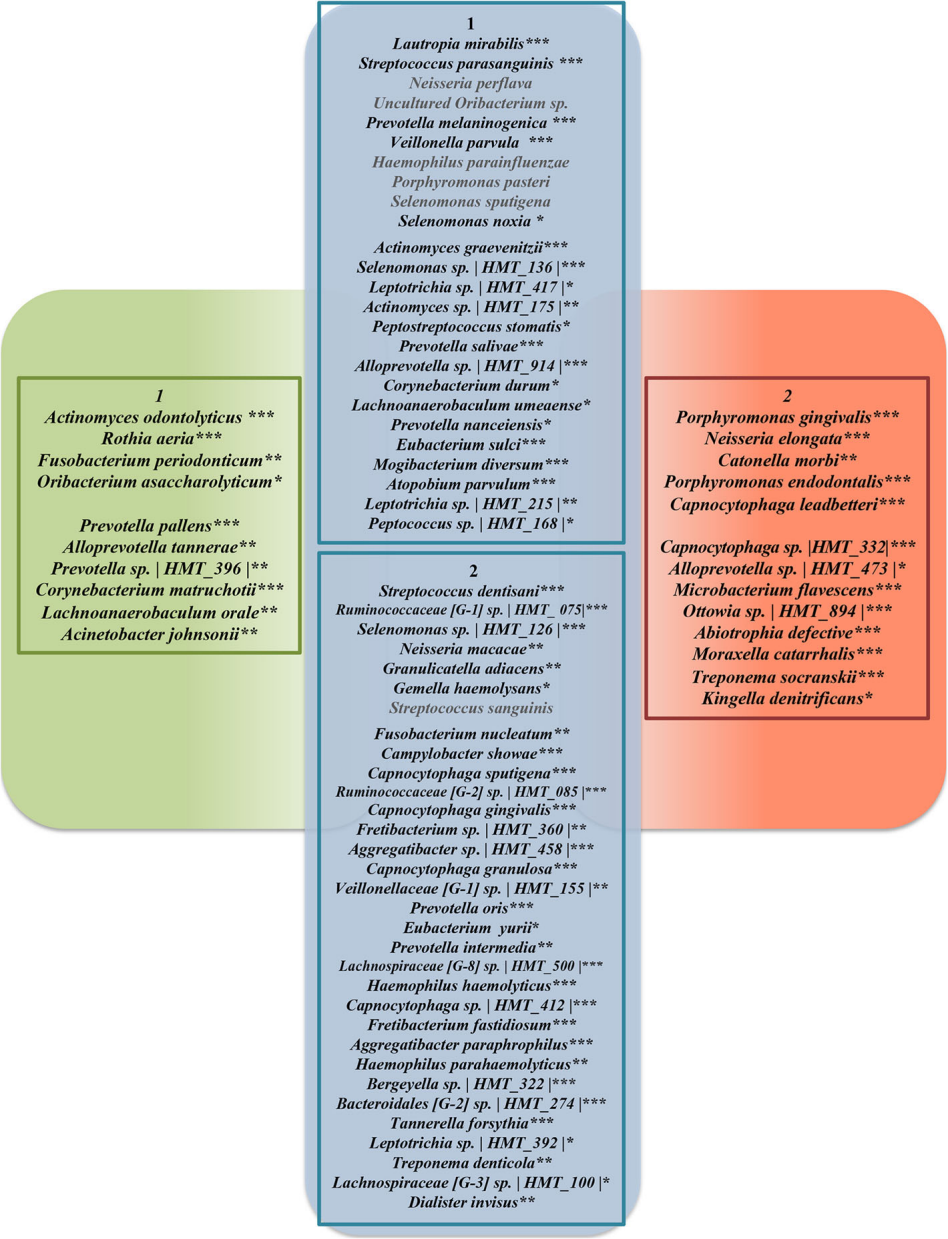
Core salivary microbiome with relative abundance > 1% or relative abundance of < 1% while presenting statistically difference in the HD patients or the healthy controls. The core microbiome was defined as species present in 75% or greater of samples. The inner box labeled with 1 indicates the species is more abundant in the healthy controls than in the HD patients. The inner box labeled with 2 indicates the species is more abundant in the HD patients than in the controls. The significance of differences between groups was assessed using Mann-Whitney U-test (*P < 0.05, **P < 0.01, ***P < 0.001)

The core microbiome with average relative abundance > 1% or species with statistically difference were charted in Fig. 3. Although many bacteria were prevalent in both communities, *Rothia aeria* (*P* < 0.001), *Actinomyces odontolyticus* (*P* < 0.001), *Fusobacterium periodonticum* (*P* = 0.001), *Oribacterium asaccharolyticum* (*P* = 0.033) were unique with relative abundances > 1% to the healthy group. On the contrary, *Porphyromonas gingivalis* (*P* < 0.001), *Neisseria elongate* (*P* < 0.001), *Catonella morbi* (*P* = 0.008), *Porphyromonas endodontalis* (*P* < 0.001) and *Capnocytophaga leadbetteri* (*P* < 0.001) were only detected in the HD group as core salivary microbiome with a mean relative abundance > 1%.

### Species associated with periodontal diseases

Considering CKD as a risk factor for periodontal health, we evaluated the microbes that are the important periodontal pathogens belonging to the “red complex” and “orange complex” [29]. HD patients appeared to have higher levels of periodontal pathogens except for *Prevotella nigrescens* (*P* = 0.147) (supplementary Table S4).

### Predicted microbial function

**Fig. 4:**
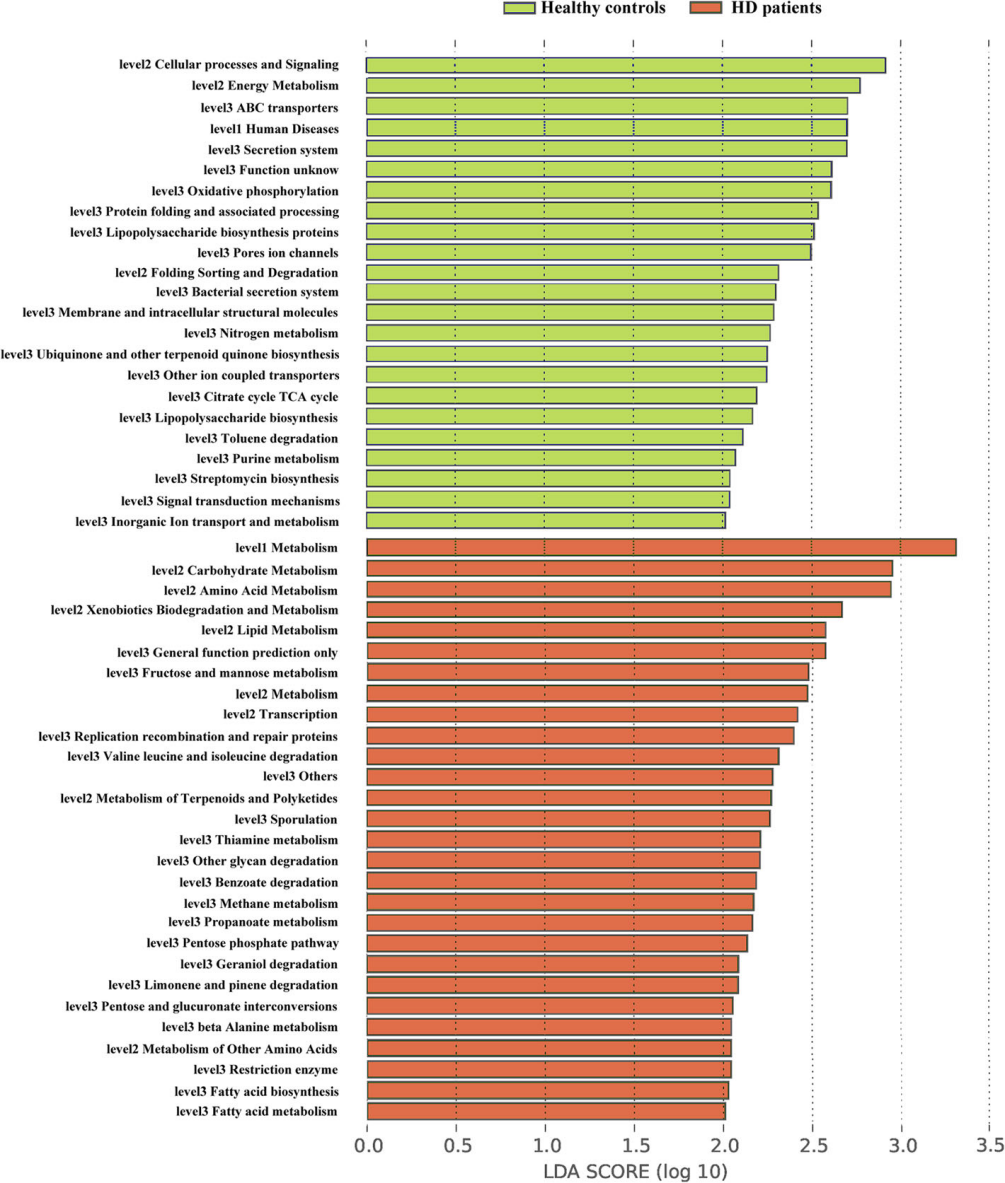
Linear discriminant analysis scores of the enriched microbial functions in the healthy controls (Green) and the HD patients (Red). LDA: Linear discriminant analysis

We generated PICRUSt to predict the gene functions in the salivary microbiome (Fig. 4). Microbes with greater relative abundances in the healthy community had functions that were most related to cellular processes and signaling (ABC transporters, secretion system and pores ion channels) and energy metabolism (oxidative phosphorylation). In contrast, the microbes in the samples of the HD patients were more likely to have functionality related to metabolism including carbohydrate metabolism (fructose and mannose metabolism), amino acid metabolism (valine leucine and isoleucine degradation, beta alanine metabolism), xenobiotics biodegradation and metabolism, as well as lipid metabolism. Moreover, microbes with the function of sporulation also increased in the HD patients.

### Associations of the salivary microbiome with duration of HD therapy and clinical indices

**Fig. 5:**
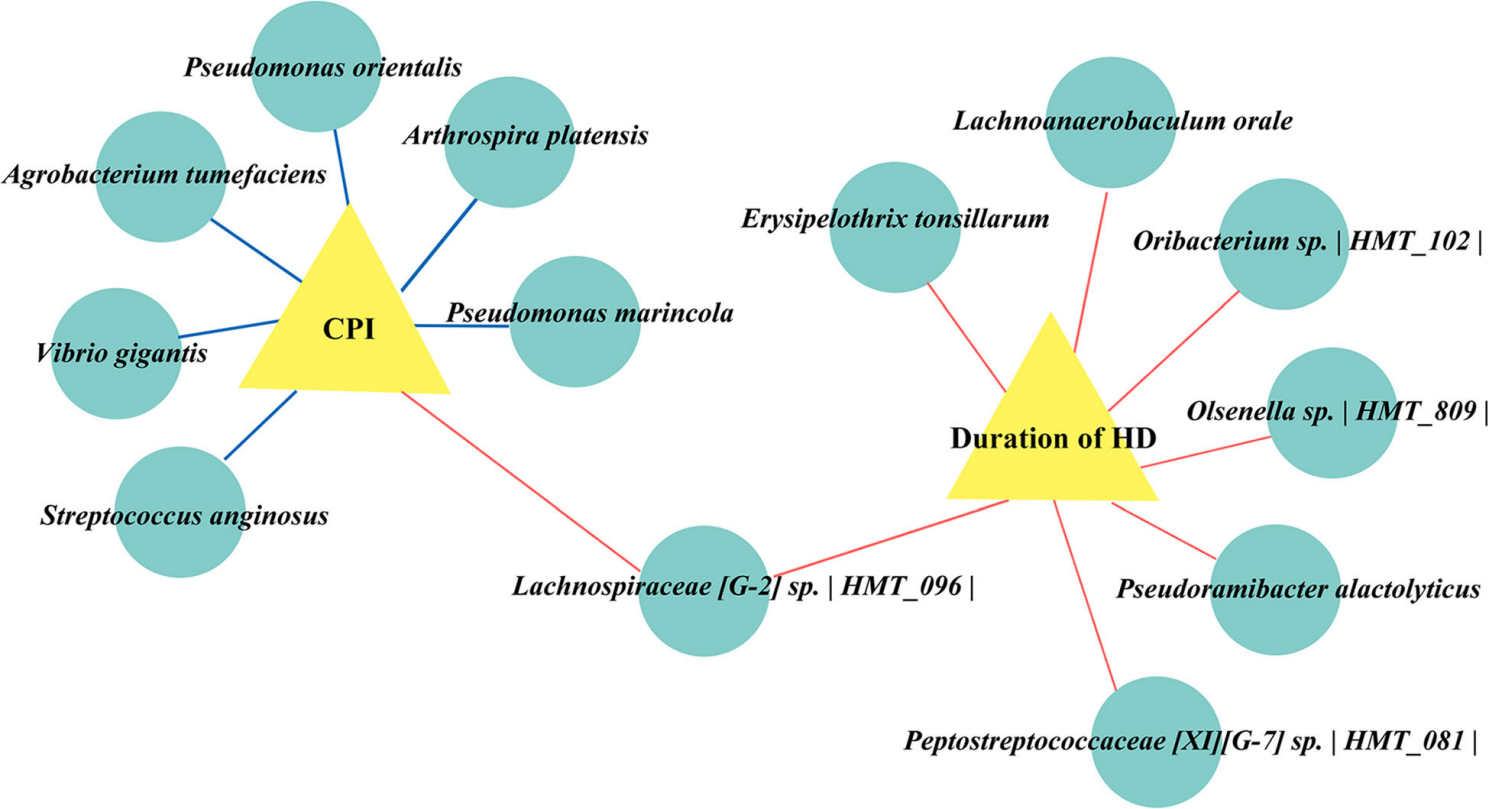
Associations of the salivary microbiotas with duration of HD and CPI index. Correlations were detected, and the indices with Spearman’s correlation (|correlation ρ| coefficients > 0.3 or 0.4, *P* < 0.05) were visualized in Cytoscape. If the correlations are negative, the connecting line is blue; if the correlation is positive, the connecting line is red. The line thickness represents the size of the correlation coefficient

Next, we performed a correlation analysis of the differential microbiota at the OTU level with the duration of HD therapy and oral clinical indices. Figure 5 showed the constructed related network (|correlation ρ| > 0.3 or 0.4, and *P* < 0.05), and the supplementary Table S5 provided the detailed description. The abundance of *Lachnospiraceae [G-2] sp.* | *HMT_096* | was positively correlated with both the duration of HD and the CPI index of the patients. *Agrobacterium tumefaciens, Pseudomonas marincola, Vibrio gigantis, Streptococcus anginosus, Pseudomonas orientalis and Arthrospira platensi* showed moderate correlations (|correlation ρ| > 0.4) with CPI index, were negatively correlated with that periodontal indicator. In addition, there were another 6 species exhibiting weak positive correlations with the duration of HD therapy (0.3 < correlation ρ < 0.4, *P* < 0.05).

To determine whether the duration of HD therapy could be a potential effect on the ESRD patients’ salivary microbiome, we divided all the patients into three groups: (i) HD1 group (*n* = 23): 3 m < duration of HD ≤1y; (ii) HD2 group (*n* = 61): 1y < duration of HD ≤5y and (iii) HD3 group (*n* = 24): duration of HD >5y. All the patients in the HD3 group were with periodontitis and the percentage of individuals with CPI 3 and CPI 4 was slightly higher than the other two groups (supplementary Table S6). The overall structure of the microbial community (supplementary Figure S1, supplementary Table S7) were not altered significantly. No significant difference was observed at the phylum level (supplementary Table S8) and among periodontal pathogens (supplementary Table S9).

## Discussion

Our previous study have found that the ESRD patients undergoing HD suffered a high prevalence and severity of periodontitis [8], further related with the survival of this population [30, 31]. Periodontitis has been identified as a novel risk factor for morbidity and mortality in patients undergoing long-term hemodialysis treatment [32–35]. Moreover, nonsurgical periodontal treatment, as a relatively simple intervention, has beneficial impact on periodontal status, circulating inflammatory markers and nutritional status in ESRD patients [36–38] that underlines the importance of oral health and efficient periodontal treatment to maintain prolong survival and quality of life in these patients. However, very few patients have regular dental care [39, 40] suggesting that more effort should be paid to provide reasonable clinical guideline for dental treatment [41] and to improve the oral health status of this population.

ESRD with HD alters the gut microbiome and may have an impact on the human oral microbiome. Alterations in microbial ecology are related to both oral and systemic diseases, including periodontitis. A recent study described the profile of the subgingival microbiome of peritoneal dialysis patients with periodontitis [42]. In this study, we aimed to investigate the microbial salivary profiles of HD patients and further explore the association between salivary microbiome, periodontal health status and duration of HD. Our finding of variations in oral health status and in salivary microbiome between the HD patients and the healthy individuals suggests that ESRD with HD obviously disturbed the salivary microbiome and periodontal health; duration of HD may further alter the abundance of some specific species, meanwhile, alterations of species may also influence the periodontal health states of the HD patients.

Saliva is a microbial reservoir that contains microbes shedding from both supra and niches. Hence, intraoral transmissions of pathogenic bacteria are likely to be mediated through saliva [43]. Salivary microbiome can enter the tract by swallowing, which may cause imbalance of the intestinal flora [23]. To elucidate the changes of salivary microbiome in ESRD patients can expand the understanding of the interactions between oral microbiome and systemic health. A number of previous studies have used saliva sample as an easy, noninvasive and inexpensive diagnostic tool to assess healthy or disease conditions over the past decades [44–46], including periodontitis [47]. Salivary changes have already been reported by previous studies [48], and understanding of the changes in salivary microbiome may present a new point to explore the relationship between ESRD undergoing HD and the other oral manifestations caused by this status, such as taste change, calculus formation and fungal infection. Moreover, previous study reported the salivary microbiome of immunoglobulin A nephropathy (IgAN) differed from that of healthy controls [49]. Inspired by these findings, in this study, we collected saliva samples from the 208 individuals.

Taxonomic differences were detected between the HD patients and the healthy controls at phylum, genus and OTU level. Besides “the others” sort, 7 of the top 10 phyla show significant difference compare with the healthy controls, indicating obvious overall changes in the compositions of the two microbial communities. Changes at phylum level in this study are consistent with previous finding, suggesting that the development of periodontal diseases is intimately linked to shift from a symbiotic dental biofilm, composed mostly of facultative anaerobic bacteria (e.g. *Streptococci* and *Actinomyces*), to a dysbiotic microbial community of anaerobic microbes belonging to the phyla essentially comprised of *Firmicutes*, *Spirochaetes*, *Synergistetes, and Bacteroidete* [50]. An increase in *Firmicutes* has also been reported in the gut microbiome of the ESRD patients during HD [22].

The core salivary microbiome of both HD patients and healthy controls comprised of 104 species (58 species shared by the two groups, while 46 differed). The data indicate that there exists a core salivary microbiome composed of microbes that are most suited to healthy individuals, whereas ESRD with HD therapy might modify this environment. *Actinomyces odontolyticus*, core microbiome of the healthy community with relative abundance > 1%, is a gram-positive facultative aerobic bacterium, which decreased significantly in the HD community. Previous studies have reported that *A. odontolyticus* was the host of the candidate phylum *Saccharibacteria*, being associated with human inflammatory mucosal diseases, thereby killed by a parasitic phase [51, 52]. By contrast, *Porphyromonas gingivalis,* core microbiome of the HD community with relative abundance > 1%, is a convincedly proposed keystone bacterium in the genesis and development of periodontitis, which was at significantly higher level in the HD group. It is worth mentioning that all the three important periodontal pathogens belonging to the “red complex” were significantly elevated in HD patients, suggesting that ESRD with HD creates a pathogen-rich oral community that could further inducing a high prevalence and a serious degree of periodontitis in this cohort. On the contrary, early study has failed to find the periodontitis-associated taxa differed between CKD and control group [53]. The reasons might include that participants were in different stages of CKD and different primers and sequencing platform were used. Notably, there is a bidirectional relationship between oral microbiome and kidney disease. Evidence suggests that periodontal pathogens and those metabolic products have adverse effects on the survival of ESKD patients who are undergoing dialysis [30]. Moreover, moderate-to-severe periodontal disease also may increase the risk of cardiovascular mortality in the dialysis patients [54]. Though nephropathies also favor the appearance of oral diseases, periodontal examination is still not part of their daily standard medical assessments. Hence, periodontitis in this population must be carefully evaluated by dental professionals, and clinical dental treatment should be taken to reverse this amendable inflammation status.

According to the LDA score, it inferred that the metagenomic pathways that were enriched in the patients undergoing HD were mainly those involved in metabolism, ranging widely from carbohydrate, amino acid and lipid. *Porphyromonas*, showing significantly higher abundance in the HD group, were amino acid degrading species, which can break down peptides and proteins into amino acids and then degrade them to produce short-chain fatty acids, sulfur compounds, ammonia, and indole/skatole, which all act as virulent influences in periodontitis [55]. The relationship between cellular processes and signaling and the healthy population is unclear.

The severity of periodontal disease slightly worsened as the HD duration prolonged. Further, we found that OTUs, positively related with the duration of HD, were mainly originated from the phyla *Firmicutes*, while OTUs being negatively related with CPI index of the HD patients were principally from the phyla *Proteobacteria*, which is similar with the higher ratio of *Firmicutes* and *Proteobacteria* in the HD community. Interestingly, *Lachnospiraceae [G-2] sp.* | *HMT_096* |, from the genera *Firmicutes*, was enriched as the extension of the duration of HD and was more abundant in the patients with severe periodontitis. The duration may not change the overall composition and structure of the salivary microbial community significantly, but contribute to changes of some specific species that may further stimulate a bad effect on the patients’ periodontal status. Considering that the available data about oral microbiota of HD patients remain limited, all these correlated nods may be helpful in finding potential periodontal pathogens or probiotic bacteria and new reasonable periodontal treatment with the HD population in the future.

## Conclusion

ESRD patients undergoing HD exhibited more severe periodontal health status, and had microbiota structural, compositional and functional differences compared with the healthy controls. Furthermore, we identified the species changed as the duration of hemodialysis prolonged. End stage renal disease changes salivary microbiome and is a risk factor for oral dysbiosis.

## Supplementary information

**Additional file 1: Table S1.** Dissimilarity Tests between the Healthy Controls and the HD Patients. **Table S2.** Relative Abundance of the Top 30 Bacterial Genera. **Table S3.** Relative abundance of the top 30 bacterial genera with significant difference between the groups. **Table S4.** Relative Abundance of the Species Associated with Diseases in the Healthy Controls and the HD Patients. **Table S5**. The detailed information of the Cytoscape analyzation. **Table S6**. Demographics and Clinical Parameters of Patients in the Three Groups. **Table S7.** Comparison of Bacterial Diversity, Richness, Observed Operational Taxonomic Units (OTUs) and Uncultured Species among the Three Groups. **Table S8**. Relative Abundance of the Top 10 Bacterial Phyla. **Table S9.** Relative Abundance of the Species Associated with Diseases in the Three Groups. **Figure S1.** PLS-DA discriminant analysis plot. PLS-DA: Partial least squares-discriminant analysis; HD1 group (*N* = 23): 3 m < duration of HD ≤1y; HD2 group (*N* = 61): 1y < duration of HD ≤5y; HD3 group (*N* = 24):5y < duration of HD.

## Data Availability

The raw sequence data and the patient data analyzed during the current study are available from the corresponding author on reasonable request.

## Abbreviations

CKD: Chronic kidney disease
CPI: Community periodontal index
DMFT: The number of decayed, missing, and filled teeth
DNA: Deoxyribonucleic acid
ESRD: Especially end stage renal disease
LDA: Linear discriminant analysis
LEfSe: Linear discriminant analysis effect size
IgAN: Immunoglobulin A nephropathy
HD: Undergoing hemodialysis
OTUs: Operational taxonomic units
PCoA: Principal co-ordinates analysis
PLS-DA: Partial least squares-discriminant analysis
PCR: Polymerase chain reaction
rRNA: Ribosomal ribonucleic acid
