# Supplementary material for: Salivary microbiome in patients undergoing hemodialysis and its associations with the duration of the dialysis
Source: BMC Nephrol. 2020 Sep 29;21:414. doi: 10.1186/s12882-020-02009-y (PMC7523083; doi:10.1186/s12882-020-02009-y)
Supplement: Supplementary file 1 — Additional file 1: Table S1. Dissimilarity Tests between the Healthy Controls and the HD Patients. Table S2. Relative Abundance of the Top 30 Bacterial Genera. Table S3. Relative abundance of the top 30 bacterial genera with significant difference between the groups. Table S4. Relative Abundance of the Species Associated with Diseases in the Healthy Controls and the HD Patients. Table S5. The detailed information of the Cytoscape analyzation. Table S6. Demographics and Clinical Parameters of Patients in the Three Groups. Table S7. Comparison of Bacterial Diversity, Richness, Observed Operational Taxonomic Units (OTUs) and Uncultured Species among the Three Groups. Table S8. Relative Abundance of the Top 10 Bacterial Phyla. Table S9. Relative Abundance of the Species Associated with Diseases in the Three Groups. Figure S1. PLS-DA discriminant analysis plot. PLS-DA: Partial least squares-discriminant analysis; HD1 group (N = 23): 3 m < duration of HD ≤1y; HD2 group (N = 61): 1y < duration of HD ≤5y; HD3 group (N = 24):5y < duration of HD. [file 12882_2020_2009_MOESM1_ESM.pdf]

Salivary microbiome in patients undergoing hemodialysis and its associations with the duration of the dialysis

Xiaobo Duan[1], Xiaolei Chen[2], Megha Gupta[3], Dutmanee Seriwatanachai[4], Hanxiao Xue[1], Qiuchan Xiong[1], Tong Xu[1], Dan Li[1], Anchun Mo[5], Xi Tang[2], Xuedong Zhou[1], Yuqing Li[1], Quan Yuan[1,3],

Author's affiliations:

[1]State Key Laboratory of Oral Diseases & National Clinical Research Centre for Oral Diseases, West China Hospital of Stomatology, Sichuan University, Chengdu, Sichuan, China

[2]Department of Nephrology, West China Hospital, Sichuan University, Chengdu, China

[3]Department of Preventive Dental Sciences, Division of Pedodontics, College of Dentistry, Al-Showajra Academic Campus, Jazan University, Gizan, Kingdom of Saudi Arabia

[4]Department of Oral Biology, Faculty of Dentistry, Mahidol University, Bangkok, Thailand

[5]Department of Oral Implantology, West China Hospital of Stomatology, Sichuan University, Chengdu, China

Corresponding author:

Quan Yuan

State Key Laboratory of Oral Diseases, West China Hospital of Stomatology, Sichuan University

Tel: +86 28 85501441; Fax: +86 28 85501441

E-mail address: yuanquan@scu.edu.cn

Yuqing Li

State Key Laboratory of Oral Diseases, West China Hospital of Stomatology, Sichuan University

No. 14, Section 3, Renmin South Road, Chengdu 610041, China.

Tel: +86 28 85502331; Fax: +86 28 85502331

E-mail address: liyuqing@scu.edu.cn

Supplementary Table S1. Dissimilarity Tests between the Healthy Controls and the HD Patients.

**Adonis**	**P-value**	**ANOSIM**	***P* value**
0.0936	0.001	0.2371	0.001

Supplementary Table S2. Relative Abundance of the Top 30 Bacterial Genera.

Gerena	Groups		*P* value
	Healthy controls (N=100)	HD Patients (N=108)	
Streptococcus	19.49±14.60	21.90±14.56	0.178
Neisseria	9.58±10.63	9.90±8.66	0.294
Lautropia	12.05±19.16	4.21±6.82	0.031
Oribacterium	6.89±11.35	5.64±9.55	0.569
Porphyromonas	3.16±3.41	4.62±4.79	0.004
Ruminococcaceae	1.63±3.48	5.74±6.91	0.000
Capnocytophaga	1.86±3.27	4.77±6.12	0.000
Prevotella [G-7]	4.12±5.00	2.05±3.12	0.000
Actinomyces	4.52±4.80	1.36±1.78	0.000
Haemophilus	2.77±3.96	2.39±2.24	0.710
Veillonellaceae	1.95±3.29	2.85±3.53	0.000
Selenomonas [G-3]	2.05±3.91	2.05±3.91	0.935
Veillonella	2.99±2.39	1.26±1.49	0.000
Granulicatella	1.42±1.46	2.37±2.26	0.000
Fusobacterium	2.03±2.17	1.64±1.33	0.641
Prevotella	1.91±2.22	1.71±1.43	0.499
Selenomonas	1.74±3.59	1.59±2.49	0.153
Gemella	1.09±1.11	1.72±2.21	0.034
Rothia	1.58±1.91	0.84±1.27	0.000
Catonella	0.87±0.99	1.36±1.45	0.003
Leptotrichia	1.33±1.63	0.89±1.10	0.027

**Gerena**	**Groups**		***P* value**
	**Healthy controls (N=100)**	**HD Patients (N=108)**	
Campylobacter	0.52±0.87	1.40±2.04	0.000
Selenomonas [G-4]	0.78±1.78	1.12±1.59	0.006
Norank f XIII	0.98±2.14	0.83±1.70	0.994
Fretibacterium	0.56±1.17	0.90±1.29	0.002
Eubacterium yurii	0.63±1.36	0.69±1.24	0.016
Alloprevotella	0.69±1.22	0.58±1.17	0.181
Treponema [G-2]	0.56±1.27	0.68±0.85	0.002
Aggregatibacter	0.25±0.45	0.88±1.15	0.000
Bergeyella	0.12±0.19	0.84±1.50	0.000

The data are presented as "Mean ± SD."

Supplementary Table S3. Relative abundance of the top 30 bacterial genera with significant difference between the groups.

Gerena	Groups		*P* value
	**Healthy controls (N=100)**	**HD Patients (N=108)**	
**Healthy group**			
Lautropia	12.05±19.16	4.21±6.82	0.031
Prevotella [G-7]	4.12±5.00	2.05±3.12	0.000
Actinomyces	4.52±4.80	1.36±1.78	0.000
Veillonella	2.99±2.39	1.26±1.49	0.000
Rothia	1.58±1.91	0.84±1.27	0.000
Leptotrichia	1.33±1.63	0.89±1.10	0.027
**HD group**			
Ruminococcaceae	1.63±3.48	5.74±6.91	0.000
Capnocytophaga	1.86±3.27	4.77±6.12	0.000
Porphyromonas	3.16±3.41	4.62±4.79	0.004
Veillonellaceae	1.95±3.29	2.85±3.53	0.000
Granulicatella	1.42±1.46	2.37±2.26	0.000
Gemella	1.09±1.11	1.72±2.21	0.034
Campylobacter	0.52±0.87	1.40±2.04	0.000
Catonella	0.87±0.99	1.36±1.45	0.003
Selenomonas [G-4]	0.78±1.78	1.12±1.59	0.006
Fretibacterium	0.56±1.17	0.90±1.29	0.002
Aggregatibacter	0.25±0.45	0.88±1.15	0.000
Bergeyella	0.12±0.19	0.84±1.50	0.000
Eubacterium yurii	0.63±1.36	0.69±1.24	0.016
Treponema [G-2]	0.56±1.27	0.68±0.85	0.002

The data are presented as “Mean ± SD”

Supplementary Table S4. Relative Abundance of the Species Associated with Diseases in the Healthy Controls and the HD Patients.

Specie	Groups		*P* value
	Healthy controls (N=100)	HD Patients (N=108)	
**The species belonging to the "Red Complex"**			
Porphyromonas gingivalis	0.68±1.68	1.61±3.73	0.000
Tannerella forsythia	0.16±0.23	0.28±0.32	0.000
Treponema denticola	0.07±0.11	0.10±0.13	0.011
**The species belonging to the "Orange Complex"**			
Campylobacter showae	0.09±0.14	0.80±1.73	0.000
Fusobacterium nucleatum	0.69±0.82	0.90±0.81	0.001
Fusobacterium periodonticum	1.34±1.73	0.74±0.94	0.001
Prevotella intermedia	0.28±0.64	0.39±0.63	0.001
Prevotella melaninogenica	0.04±0.07	0.01±0.01	0.000
Prevotella nigrescens	0.05±0.12	0.10±0.22	0.147

The data are presented as "Mean ± SD"

Supplementary Table S5. The detailed information of the Cytoscape analyzation.

OTU		Phylum	Family	*P* value	correlation r
**Positive correlations**					
OTU1433	*Lachnospiraceae [G-2] sp. |HMT_096|*	*Firmicutes*	*Lachnospiraceae [XIV]*	0.0128	0.31867
OTU1433	*Lachnospiraceae [G-2] sp. |HMT_096|*	*Firmicutes*	*Lachnospiraceae [XIV]*	0.0001	0.33249
OTU27	*Erysipelothrix tonsillarum*	*Firmicutes*	*Erysipelotrichaceae*	0.0015	0.30225
OTU902	*Lachnoanaerobaculum orale*	*Firmicutes*	*Lachnospiraceae [XIV]*	0.0003	0.33813
OTU505	*Oribacterium sp. |HMT_102|*	*Firmicutes*	*Lachnospiraceae [XIV]*	0.0010	0.31225
OTU17	*Olsenella sp. |HMT_809|*	*Actinobacteria*	*Atopobiaceae*	0.0020	0.30447
OTU788	*Peptostreptococcaceae [XI][G-7] sp. |HMT_081|*	*Firmicutes*	*Peptostreptococcaceae [XI]*	0.0021	0.30173
OTU914	*Pseudoramibacter alactolyticus*	*Firmicutes*	*Eubacteriaceae*	0.0020	0.30372
**Negative correlations**					
OTU1301	*Vibrio gigantis*	*Proteobacteria*	*Vibrionaceae*	$<$0.0001	-0.40778
OTU1017	*Streptococcus anginosus*	*Firmicutes*	*Streptococcaceae*	$<$0.0001	-0.40319
OTU1009	*Pseudomonas marincola*	*Proteobacteria*	*Pseudomonadaceae*	$<$0.0001	-0.40864
OTU1160	*Agrobacterium tumefaciens*	*Proteobacteria*	*Rhizobiaceae*	$<$0.0001	-0.41443
OTU1367	*Pseudomonas orientalis*	*Proteobacteria*	*Pseudomonadaceae*	$<$0.0001	-0.40259
OTU1183	*Arthrospira platensis*	*Cyanobacteria*	*Oscillatoriales*	$<$0.0001	-0.40193

Supplementary Table S6. Demographics and Clinical Parameters of Patients in the Three Groups.

Characteristics	Groups			*P* value
	HD1 (N=23)	HD2 (N=61)	HD3 (N=24)	
Age [years] (Mean ± SD)	44.61±13.23	46.24±13.45	47.58±13.53	0.698
Male/Female	11/12	35/26	16/8	0.426
BMI [kg/m^2] (Mean ± SD)	21.96±4.10 (17.21-31.45)	22.83±3.65 (16.22-36.33)	21.93±3.23	0.304
Length of HD therapy (Mean ± SD)	0.78±0.29-	3.17±0.94	8.33±2.04	-
CPI[c]				0.299
0	0 (0.0%)	0 (0.0%)	0 (0.0%)	-
1	1 (4.3%)	0 (0.0%)	0 (0.0%)	0.430
2	4 (17.4%)	4 (6.5%)	3 (12.5%)	0.311
3	10 (43.5%)	27 (44.3%)	8 (33.3%)	0.694
4	8 (34.8%)	30 (49.2%)	13 (54.2%)	0.382
X	0 (0.0%)	0 (0.0%)	0 (0.0%)	-
Number of Decayed teeth (Mean, range)	0.70, 0-3	0.97, 0-5	0.71, 0-3	0.707
Number of Missing teeth (Mean, range)	0.96, 0-5	1.05, 0-15	1.35, 0-15	0.632
Number of Filled teeth (Mean, range)	0.39, 0-4	0.43, 0-3	0.33, 0-3	0.925

"-" = vacancy.

CPI: The data are presented as n (%).The categories of CPI were defined as: 0 = healthy gingiva; 1 = bleeding observed directly or by using a mouth mirror after probing; 2 = calculus detected during probing but with all of the black bands on the probe visible; 3 = pocket of 4 to 5 mm (gingival margin within the black band on the probe); 4 = pocket of 6 mm (black band on the probe was not visible); X = excluded sextant (<2 teeth present)

Supplementary Table S7. Comparison of Bacterial Diversity, Richness, Observed Operational Taxonomic Units (OTUs) and Uncultured Species among the Three Groups.

	Groups			*P* value
	**HD1 (N=23)**	**HD2 (N=61)**	**HD3 (N=24)**	
**Chao**	263.44±46.04	257.10±37.52	258.00±46.67	0.826
**Shannon**	3.51±0.33	3.40±0.54	3.40±0.44	0.829
**Observed OTUs**	221.30±39.45	217.87±33.39	212.67±38.68	0.639
**Uncultured species**	21.93±10.00-	23.63±14.04	23.96±15.15	0.896

The data are presented as "Mean ± SD"

Supplementary Table S8. Relative Abundance of the Top 10 Bacterial Phyla.

	Groups			*P* value
	HD1 (N=23)	HD2 (N=61)	HD3 (N=24)	
Firmicutes	51.04±9.24	53.36±15.04	53.85±14.93	0.749
Proteobacteria	19.70±8.55	19.66±11.64	20.74±11.84	0.917
Bacteroidetes	18.58±8.03	16.26±10.74	15.66±9.02	0.545
Actinobacteria	3.47±2.60	4.26±3.71	3.04±2.63	0.510
Fusobacteria	2.88±2.17	2.46±1.88	2.61±1.91	0.770
Synergistetes	1.08±1.33	0.83±1.06	1.09±1.89	0.594
Tenericutes	0.93±1.08	0.84±1.27	0.72±1.09	0.158
Spirochaetae	0.73±1.00	0.78±0.94	0.86±0.99	0.819
Gracilibacteria	0.93±2.38	0.69±0.95	0.88±2.34	0.758
Saccharibacteria	0.35±0.39	0.51±1.37	0.26±0.29	0.431
**Others**	0.30±0.31	0.35±0.30	0.28±0.26	0.439

The data are presented as “Mean ± SD”

Supplementary Table S9. Relative Abundance of the Species Associated with Diseases in the Three Groups.

Specie	Groups			*P* value
	HD1 (N=23)	HD2 (N=61)	HD3 (N=24)	
**The species belonging to the "Red Complex"**				
Porphyromonas gingivalis	1.12±2.02	1.86±4.03	1.45±1.85	0.269
Tannerella forsythia	0.28±0.25	0.28±0.33	0.27±0.35	0.724
Treponema denticola	0.12±0.20	0.09±0.11	0.10±0.12	0.902
**The species belonging to the "Orange Complex"**				
Campylobacter showae	0.59±0.74	0.97±2.22	0.57±0.61	0.953
Fusobacterium nucleatum	0.97±0.79	0.95±0.94	0.71±0.37	0.727
Fusobacterium periodonticum	1.06±1.49	0.70±0.77	0.54±0.59	0.517
Prevotella intermedia	0.29±0.59	0.42±0.61	0.42±0.73	0.705
Prevotella melaninogenica	0.01±0.03	0.01±0.01	0.01±0.01	0.633
Prevotella nigrescens	0.07±0.12	0.08±0.15	0.16±0.39	0.634

The data are presented as "Mean ± SD"

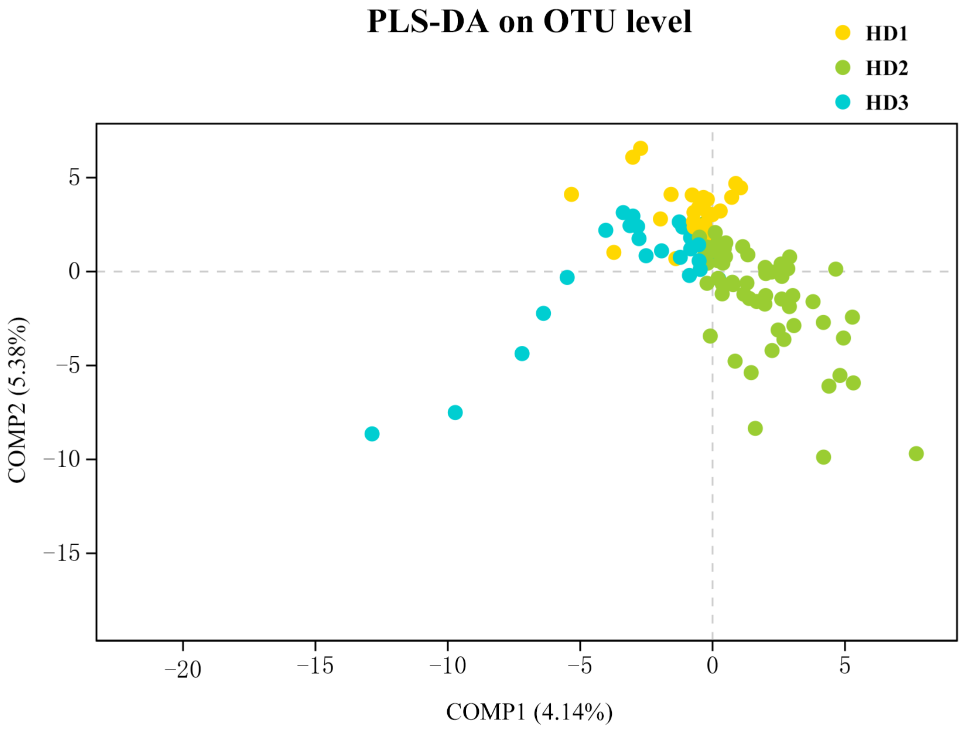

Supplementary Figure S1. PLS-DA discriminant analysis plot. PLS-DA: Partial least squares-discriminant analysis; HD1 group (N=23): 3m< duration of HD ≤1y; HD2 group (N=61): 1y< duration of HD ≤5y; HD3 group (N=24):5y< duration of HD.
